# Interleukin-6 Induces DEC1, Promotes DEC1 Interaction with RXRα and Suppresses the Expression of PXR, CAR and Their Target Genes

**DOI:** 10.3389/fphar.2017.00866

**Published:** 2017-11-28

**Authors:** Rui Ning, Yunran Zhan, Shuangcheng He, Jinhua Hu, Zhu Zhu, Gang Hu, Bingfang Yan, Jian Yang, Wei Liu

**Affiliations:** ^1^Department of Pharmacology, Nanjing Medical University, Nanjing, China; ^2^Pharmaceutical Sciences, Center for Pharmacogenomics and Molecular Therapy, University of Rhode Island, Kingston, RI, United States

**Keywords:** DEC1, interleukin-6, PXR/CAR, target genes, hepatocytes

## Abstract

Inflammatory burden is a primary cellular event in many liver diseases, and the overall capacity of drug elimination is decreased. PXR (pregnane X receptor) and CAR (constitutive androstane receptor) are two master regulators of genes encoding drug-metabolizing enzymes and transporters. DEC1 (differentiated embryonic chondrocyte-expressed gene 1) is a ligand-independent transcription factor and reportedly is induced by many inflammatory cytokines including IL-6. In this study, we used primary hepatocytes (human and mouse) as well as HepG2 cell line and demonstrated that IL-6 increased DEC1 expression and decreased the expressions of PXR, CAR, and their target genes. Overexpression of DEC1 had similar effect as IL-6 on the expression of these genes, and knockdown of DEC1 reversed their downregulation by IL-6. Interestingly, neither IL-6 nor DEC1 altered the expression of RXRα, a common dimerization partner for many nuclear receptors including PXR and CAR. Instead, DEC1 was found to interact with RXRα and IL-6 enhanced the interaction. These results conclude that DEC1 uses diverse mechanisms of action and supports IL-6 downregulation of drug-elimination genes and their regulators.

## Introduction

Pregnane X receptor (PXR, NR1I2) and CAR (NR1I3) are liver-enriched nuclear receptors that promiscuously recognize a wide range of structurally diverse compounds, particularly xenobiotics. These receptors are recognized as master regulators of genes involved in the elimination of therapeutic agents and other xenobiotics. PXR and CAR share ligand specificity as well as gene targets they regulate. For examples, phenobarbital has been shown to activate both CAR and PXR (Braeuning et al., 2014; Elcombe et al., 2014). CITCO [6-(4-chlorophenyl)-imidazo(2,1-b)thiazole-5-carbaldehyde], a potent CAR agonist, activates PXR at a high concentration (Maglich et al., 2003). Likewise, both CAR and PXR have been shown to support the induction of CYP2B6, CYP3A4, and MDR1 (Blumberg et al., 1998; Honkakoski et al., 1998; Goodwin et al., 1999). Normally, PXR and CAR are tethered in the cytoplasm (Squires et al., 2004; Qatanani and Moore, 2005). In the presence of a ligand, PXR and CAR translocate to the nucleus (Rencurel et al., 2005; Vluggens and Reddy, 2012; Jia et al., 2014). It has been well established that PXR and CAR predominately form dimers with RXR α (NR2B1) and deliver potent transactivation activity.

Human DEC1, a ligand-independent transcription factor, has been shown to support a wide range of cellular events such as cell differentiation, apoptosis, and senescence (Li et al., 2006; Qian et al., 2012, 2014). Also, DEC1 is a core circadian gene (Iizuka and Horikawa, 2008; Hu et al., 2015) and the expression of DEC1 is increased in response to environmental stimuli such as light pulses, hypoxia, and cytokines, including tumor necrosis factor-α (TNFα), interleukin-1b (IL-1b), and IL-6 (Miyazaki et al., 2002, 2010; Ivanova et al., 2004; Mao et al., 2012). IL-6 is known to downregulate the expression of a variety of drug-metabolizing enzymes including CYP3A4 (Miyazaki et al., 2002, 2010; Yang et al., 2010; Mao et al., 2012) and CYP2B6 as well as their mouse counterparts (Miyazaki et al., 2002, 2010; Yang et al., 2010; Ghose et al., 2011; Mao et al., 2012). DEC1 has been shown to repress the transactivation of the RXRα heterodimers with nuclear receptors, such as LXR, FXR, VDR, and RAR by the activity of their promoters (Cho et al., 2009; Knackstedt et al., 2013; Hoeke et al., 2014).

This study was conducted to determine the role of DEC1 in IL-6 regulated expression of PXR, CAR and their targets CYP2B6, CYP3A4, and MDR1. In both human primary hepatocytes and the hepatocellular carcinoma cell line HepG2, IL-6 increased DEC1 expression and decreased expressions of PXR, CAR, and their target genes CYP3A4, MDR1, and CYP2B6. Overexpression of DEC1 had similar effect as IL-6 on the expression of these genes, and knockdown of DEC1 reversed IL-6 effect. Similar observations were made in mouse primary hepatocytes. Interestingly, neither IL-6 nor DEC1 altered the expression of RXRα. Instead, DEC1 was found to interact with RXRα and IL-6 enhanced the interaction. These results conclude that DEC1 uses diverse mechanisms of action and supports IL-6 downregulation of drug-elimination genes and their regulators.

## Materials and Methods

### Materials

IL-6 was purchased from R&D Systems (Minneapolis, MN, United States). DMEM was purchased from Invitrogen (Carlsbad, CA, United States). FBS was purchased from Hyclone (Logan, UT, United States). GeneJet^TM^ DNA Vitro Transfection Reagent (VerII) was purchased from SignaGen Laboratories (Gaithersburg, MD, United States). SYBR Premix Ex Taq^TM^ kit was purchased from the Biotechnology Limited Company (TaKaRa, Japan). Anti-SHARP2 (STAR13) was purchased from GeneTex (GeneTex, MI, United States). GAPDH antibody was purchased from Bioworld Technology, Inc. (Louis Park, MN, United States). CYP3A11 and STRA13 antibodies were from Abgent (San Diego, CA, United States). The CYP2B10 antibody was purchased from Millipore (Billerica, MA, United States). Antibodies against PXR, mPXR, CAR, RXRa, CYP3A4, CYP2B6, and MDR1 were obtained from Abcam (Burlingame, CA, United States). Polyclonal antibody against DEC1 was described elsewhere (Li et al., 2006). Protein A/G Plus-Sepharose beads and hRXRα constructs were obtained from Santa Cruz Biotechnology (Santa Cruz, CA, United States). Goat anti-rabbit-IgG conjugated with horseradish peroxidase, BCA assay kit and ECL substrate were purchased from Pierce (Rockford, IL, United States). The P450-Glo^TM^ CYP3A4 assay kit was purchased from Promega (Madison, WI, United States). DEC1 shRNA constructs were purchased from Santa Cruz (Santa Cruz, CA, United States). The DEC1 expression construct and the DEC1 mutant constructs were described previously (Mao et al., 2012). Other chemicals and constructs without statements were all purchased from Sigma–Aldrich (St. Louis, MO, United States).

### Cell Culture and Treatment

Plated primary human hepatocytes (in six-well plates) were obtained from the Liver Tissues Procurement and Distribution System (University of Minnesota, Minneapolis, MN, United States) or the commercial source CellzDirect (Pittsboro, NC, United States). In total, nine donors were included: four males (21–59 years old) and five females (35–72 years old). Among the donors, six were white and three were African American. None of them was a smoker. Upon arrival, media were immediately replaced with rich Williams’ E medium containing insulin-transferrin-selenium supplement and 100 U/ml penicillin, 10 μg/ml streptomycin (Yang and Yan, 2007). After being incubated at 37°C with 5% CO_2_ for 24 h, hepatocytes were treated with 20 ng/ml IL-6 for another 24 h.

HepG2 cells were obtained from the Cell Bank of Shanghai, Institute of Biochemistry and Cell Biology, Chinese Academy of Sciences. Cells were cultured in DMEM supplemented with 5% FBS and incubated at 37°C in a humidified environment containing 5% CO_2_. Cells were seeded at a density of 6 × 10^5^ cells per well overnight, and then the cells were treated with IL-6 in a 1% serum-reduced medium for 24 h.

ICR mice, male, 18–22 g were obtained from the experimental animal center of Nanjing Medical University. Primary mouse hepatocytes were isolated from mouse livers using two-step perfusion (Feng et al., 2012). Cells were cultured in DMEM supplemented with 10% FBS and seeded into collagen-coated six-well plates and incubated at 37°C in 5% CO_2_ for 4 h to allow attachment. After 2 days, with a medium change every 24 h, the cells were treated with IL-6 in a 1% serum-reduced medium for 24 h. The samples on this study were conducted in accordance with the guidelines of the Institute for Laboratory Animal Research of Nanjing Medical University. The protocols were approved by the Committee on the Ethics of Animal Experiments of Nanjing Medical University (Permit Number: 14030106).

### Enzyme Activity Assay (CYP3A4)

HepG2 cells were seeded in a 48-well plate at a density of 1 × 10^5^ cells per well overnight and were treated with 10 ng/mlIL-6 or transfected with FlagDEC1 (800 ng) for 24 h. The activity of CYP3A4 was determined by using the P450-Glo^TM^ CYP3A4 assay kit according to the manufacturer’s instructions. Briefly, cell medium was replaced by a mixture of 150 μl DMEM and 150 μl Luciferin-IPA. Cells were then incubated at 37°C at 5% CO_2_ for 1 h. Next, 50 μl of culture medium from each well was transferred to a 96-well opaque white luminometer plate, and 50 μl of Luciferin Detection Reagent was added to initiate the luminescence. Luminescence was quantified by using the Spectral Scanning Multimode Reader (Thermo Scientific).

### Intracellular Rhodamine 123 Accumulation Assay

Efflux activity of MDR1 was evaluated by intracellular accumulation assay of a well-established substrate for MDR1, Rho123. HepG2 cells were cultured in 6-well plates and treated with either IL-6 (10 ng/ml) or the same volume of PBS for 24 h. Or cells were transfected with either the vector or FlagDEC1 (800 ng) for 48 h. Cells were then rinsed with PBS and resuspended in DMEM supplemented with 5 μg/ml Rhodamine (Rho123) and incubated in the absence of light at 37°C, in a humidified atmosphere of 5% CO_2_ for 1.5 h. After being washed twice with ice-cold PBS, cells were analyzed immediately by flow cytometry (BD FACSCalibur with Cell quest software). Analysis was gated to exclude cell debris and clumps on the basis of forward and side light-scatter and was based on the acquisition of data from 10,000 cells. Log fluorescence data were collected and displayed as single parameter histograms.

### Knockdown and Overexpression Experiments

To define the role of DEC1 in the IL-6-mediated down-regulation of PXR and CAR, the expression of DEC1 was regulated by shRNA and overexpression. HepG2 cells were plated in 6-well plates at a density of 6 × 10^5^ cells per well overnight. Transfection was conducted using GeneJet^TM^ (Ver II). For experiments involving shRNA, cells were transfected with a DEC1 shRNA construct (800 ng per well). After a 72 h incubation period that included two medium changes, the transfected cells were treated with PBS or 10 ng/ml IL-6 for 24 h, and expression of PXR, CAR, and DEC1 was assessed using Western blotting. For overexpression, cells were transfected with the human DEC1 construct (800 ng per well) or the corresponding vector. After a 48 h incubation period, the cells were treated with PBS or 10 ng/ml IL-6 for 24 h, and the expression of PXR, CAR, and DEC1 was quantified by using Western blotting.

### Transient Co-transfection Experiment (Luciferase Assay)

HepG2 cells were seeded in a 48-well plate at a density of 1 × 10^5^ cells per well. Transfection mixtures contained 50 ng PXR-1289-Luc reporter plasmid and 5 ng of pRL-TK luciferase plasmids. Cells were transfected for 12 h and then treated with 10 ng/ml IL-6 or PBS for an additional 24 h. To detect whether DEC1 can mimic the effect of IL-6 on PXR reporter, cells were transfected with mixtures containing 50 ng of PXR-1289-Luc and various doses of FlagDEC1 (1–50 ng) or DEC1-M (50 ng) along with 5 ng of Null-Renilla reniformis luciferase plasmid by using GeneJet^TM^ (Ver II).The reporter activity was assayed by using a Dual-Luciferase Reporter Assay System. The firefly luciferase activity, which was indicative of the reporter activity, was activated by mixing lysates (10 μl) with Luciferase Assay Reagent II. Firefly luminescence was quenched, while Renilla luminescence was simultaneously activated by the addition of Stop and Glo Reagent to each sample. The firefly luminescence signal was normalized based on the Renilla luminescence signal. The ratio of normalized luciferase activity was calculated by that luminescence values obtained from cells treated with IL-6 or transfected with DEC1 over luminescence values obtained from cells treated with PBS or transfected with the vector.

### Real-Time Polymerase Chain Reaction

Total RNA was isolated by using Trizol according to the manufacturer’s instruction. First-strand cDNA was synthesized using total RNA (1 μg) and the following cycling parameters: 70°C for 5 min, 42°C for 60 min, and 95°C for 10 min using oligo dT 12-18 primer and MLV reverse transcriptase. The TaqMan assay identification numbers were as follows: DEC1, Hs00186419_m1; PXR, Hs00243666_m1; CAR, Hs00231959_m1; RXRα, Hs00172565_m1;CYP3A4, Hs00604506_m1; CYP2B6, Hs00167937_g1; MDR1, Hs00184491_m1; and GAPDH, 4352934E. For the SYBR green assay, the primer sequences used for PCR were as follows: CYP3A4 forward, TCAATAACAGTCTTTCCATTCCTCAT and reverse, CTTCGAGGCGACTTTCTTTCA; CYP2B6 forward, GCACTCCTCACAGGACTCTTG and reverse, CCCAGGTGTACCGTGAAGAC; MDR1 forward, GTCCCAGGAGCCCATCCT and reverse, CCCGGCTGTTGTCTCCATA; PXR forward, CGAGCTCCGCAGCATCA and reverse, TGTATGTCCTGGATGCGCA; CAR forward, CAGGGTTCCAGTACGAGTT and reverse, AGCCGAGACTGTTGTTCC; RXRαforward, CCAGGTGAACTCTTCGTCC and reverse, TGTCTCGGCAGGTGTAGGT; DEC1 forward, GGCGGGGAATAAAACGGAGCGA and reverse, CCTCACGGGCACAAGTCTGGAA. PCR amplification and quantification were conducted using the Applied Biosystems 7900 or 7300 and the following conditions: 50°C for 2 min, 95°C for 10 min, followed by 40 cycles of 95°C for 15 s and 60°C for 1 min. Gene expression changes were calculated by the comparative *C*t method, and the values were normalized to the endogenous reference gene GAPDH.

### Extraction of Subcellular Fractions

At the end of treatment, cells were harvested and washed with ice-cold PBS. Cytoplasm and nucleus fractions were extracted by using a nuclei isolation kit (KeyGen Biotech, Nanjing, China). Isolated cytoplasm and nucleus fractions were checked for purity by Western blotting analysis of the respective markers for cytoplasmic or nuclear components. GAPDH was used as internal control of cytoplasm. H3 was used as internal control of nucleus.

### Western Blotting

HepG2 cell lysates (normally 80 ∼ 100 μg) and primary mouse hepatocytes lysates (40 μg) were resolved by 7.5% SDS-polyacrylamide gel-electrophoresis. The blots were incubated

by using antibodies against CYP3A4 (1:2000), CYP3A11 (1:1000), CYP2B6 (1:2000), CYP2B10 (1:3000), DEC1 (1:5000), STRA13 (1:2000), PXR (1:2000), CAR (mCAR) (1:2000), RXRα (1:2000), MDR1 (P-gp) (1:2000), and GAPDH (1:3000) (**Supplementary Table S1**). Protein levels were quantified by density analysis through using Image Analysis software (NIH) and expressed as interest protein/internal control. Protein concentrations were determined by using the BCA assay which based on the albumin standard.

### Immunofluorescence Analysis

HepG2 cells were seeded at a density of 2 × 10^5^ cells on glass bottom dishes and got corresponding treatment. Then, the cells were fixed with 4% paraformaldehyde in phosphate buffer (pH 7.4) for 20 min at room temperature. After washing cells with PBS and blocking with 5% bovine serum albumin (BSA) for 1 h, the cells were incubated with the anti-DEC1 antibody at 4°C overnight. Tetramethylrhodamine (TMR) labeled anti-rabbit IgG antibody was added to the cells and incubated at 37°C for 1 h. Cells were examined under a laser scanning confocal microscope (ZEISS LSM 790).

### Dual-Labeled Immunofluorescence Analyses

HepG2 cells were plated at a density of 2 × 10^5^ cells per well in glass bottom dishes overnight. Then, they were transfected with vector alone, FlagDEC1 (800 ng), or FlagDEC1 (800 ng) and RXRα (800 ng) for 24 h. Vector plasmid was used to equalize the amount of plasmid DNA for each transfection. The transfected cells were washed three times with PBS, then treated with the primary antibodies (either DEC1 1:400 or RXRα 1:200). The secondary antibodies were added before treatment with Fluoroshield with DAPI. Images were taken using a confocal microscope (488 and 543 nm wavelength). All images were acquired using a 63× magnification and an oil immersion objective on a Laser scanning confocal microscope.

### *In Vivo* Protein–Protein Cross-Linking by Formaldehyde

HepG2 cells were cultured in 6-well plates and transfected with FlagDEC1 (800 ng), FlagDEC1 (800 ng) + RXRα (800 ng) or vector plasmid. Then, after 48 h, cells were fixed using 4% formaldehyde at room temperature for 15 min. Cross-linking was terminated by the addition of glycine to a final concentration of 0.1 M. Cells were harvested in ice-cold PBS, and the cell pellet was resuspended in ice-cold lysis buffer, followed by centrifugation at 4°C for 10 min. Immune complexes were analyzed by Western blotting using either anti-DEC1 or anti-RXRα antibodies.

### Co-immunoprecipitation (Co-IP) Assays

HepG2 cells were seeded at a density of 3 × 10^6^ cells/dish (50 ml) overnight and treated with IL-6 (10 ng/ml) or the same volume of PBS for 24 h or were transfected with FlagDEC1 (2 μg) or the same amount of FlagCMV2 for 48 h. Total cell lysates were prepared using an extraction kit. Cell extracts (500 μg) were incubated with 3.5 μg of anti-DEC1 antibody at 4°C overnight. Immune complexes were precipitated using 30 μl of Protein A/G Plus-Sepharose beads at 4°C for 4 h. The beads were washed eight times with ice-cold wash buffer (50 mM Tris/HCl, pH 7.4 and 150 mM NaCl). The precipitates were analyzed for the presence of RXRα by Western blotting analysis using an anti-RXRα antibody. The samples (5 μg) before immunoprecipitation were also analyzed by Western blotting using anti-DEC1 and anti-RXRα antibodies.

### Statistical Analysis

Data were presented as the mean ± SD. Standard deviations of the mean were calculated by using values from at least three independent experiments. Statistical analysis was performed using SAS soft ware version 9.1 (SAS Institute, Cary, NC, United States). The significant difference was claimed at *p* < 0.05 based on one-way or two-way analysis of variance followed by Duncan’s multiple comparison tests.

## Results

### IL-6 Decreases PXR, CAR and Their Target Genes But Increases DEC1 in Primary Human Hepatocytes and HepG2 Cells

**FIGURE 1:**
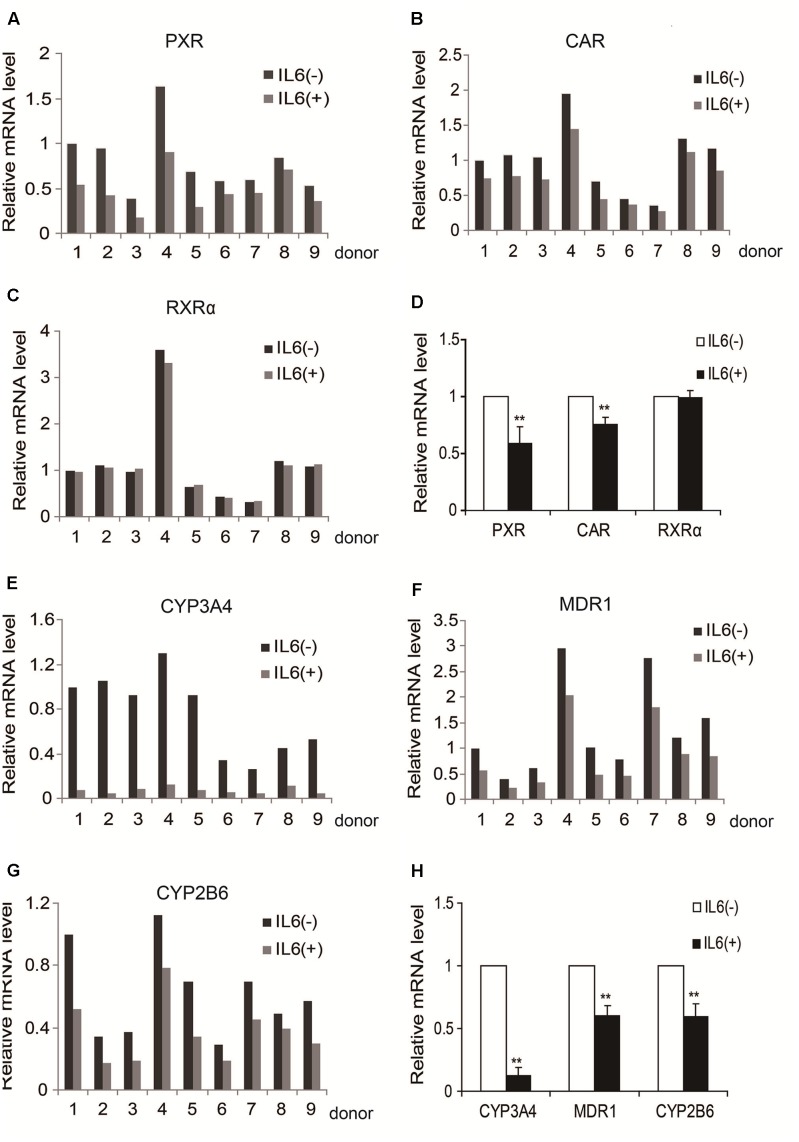
IL-6 decreases the expression of PXR, CAR and their target genes in primary human hepatocytes (nine individuals). **(A–D)** Human primary hepatocytes from nine donors were treated with IL-6 (20 ng/ml) or PBS for 24 h. Total RNA was isolated and subjected to quantitative reverse transcription-polymerase chain reaction analysis for the mRNA levels of PXR, CAR, and RXRα by TaqMan probes. **(E–H)** Then the mRNA level of their target genes, CYP3A4, MDR1, and CYP2B6, were detected by TaqMan probes. The signals from each target were normalized based on the signal from glyceraldehyde-3-phosphate dehydrogenase (GAPDH). Data are expressed as mean ± SD. ^∗∗^*p* < 0.01 vs. PBS.

We previously reported that IL-6 downregulated PXR and CYP3A4 in primary human hepatocytes (Yang et al., 2010; Mao et al., 2012). We extended these observations and tested whether IL-6 downregulates MDR1, CYP2B6, CAR, and RXRα. As shown in **Figure 1**, treatment with IL-6 decreased the mRNA expression of PXR and CAR (**Figures 1A,B,D**) but not RXRα (**Figures 1C,D**). Consistent with the downregulation of PXR and CAR, the expression of their targets, CYP3A4, MDR1, and CYP2B6, decreased with CYP3A4 being the most (**Figures 1E–H**).

**FIGURE 2:**
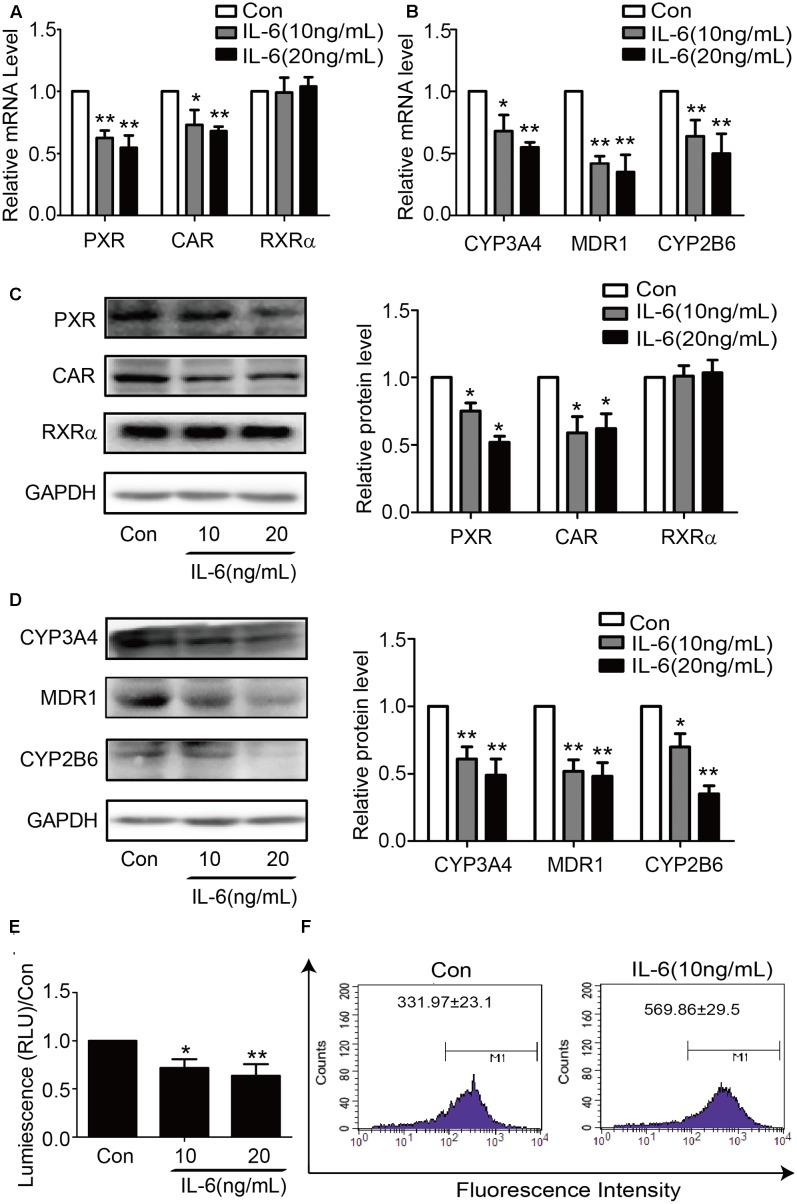
IL-6 decreases the expression of PXR, CAR and their target genes in HepG2 cells. HepG2 cells were treated with IL-6 (10 ng/ml) or PBS for 24 h, and the mRNA and protein levels of PXR, CAR, RXRα, CYP3A4, MDR1, and CYP2B6 were detected by qRT-PCR (SYBR green) **(A,B)** and Western blotting **(C,D)**. **(E)** Repression of CYP3A4 catalytic level induced by IL-6 with indicated concentration. **(F)** Decrease of the efflux activity MDR1 induced by IL-6. HepG2 cells incubated with 5 μg/ml Rho123. Intracellular Rho123 accumulation was analyzed by flow cytometry. Representative histogram plots with the indicated treatments are provided, with fluorescence intensity shown as the mean ± SD. All the experiments were repeated at least three times, ^∗^*p* < 0.05, ^∗∗^*p* < 0.01 vs. Con.

To determine whether similar responding profile occurs in hepatic cell line, HepG2 cells were treated with IL-6 and the mRNA level for these genes determined. Consistent with the results in primary hepatocytes, the mRNA level of all genes except RXRα significantly decreased in IL-6 treated cells (**Figures 2A,B**). Similar changes were detected on the level of protein expression (**Figures 2C,D**). To ascertain the functional link, HepG2 cells were treated with IL-6, and cell lysates were prepared, and the activity of CYP3A4 activity was determined. As shown in **Figure 2E**, the activity of CYP3A4 was significantly decreased in cells treated with 10 or 20 ng/ml IL-6. Likewise, the efflux activity of MDR1 was decreased as well (**Figure 2F**).

**FIGURE 3:**
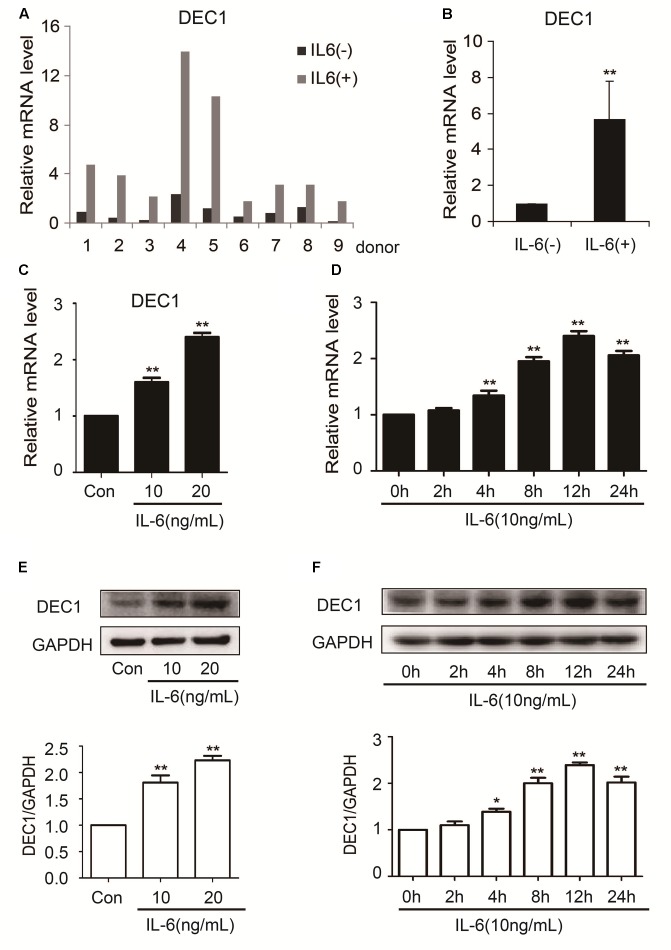
IL-6 increases DEC1 expression both in primary human hepatocytes and HepG2 cells. **(A,B)** Human primary hepatocytes from nine donors were treated withIL-6 (20 ng/ml) or PBS for 24 h. Total RNA was isolated and subjected to quantitative reverse transcription-polymerase chain reaction analysis for the mRNA level of DEC1 by TaqMan probes. The signals from each target were normalized based on the signal from glyceraldehyde-3-phosphate dehydrogenase (GAPDH). HepG2 cells were treated with IL-6 (10 and 20 ng/ml) or PBS for 24 h, or were treated with IL-6 (10 ng/ml) for 2, 4, 8, 12, and 24 h, respectively, and the DEC1 expression at both mRNA and protein levels were analyzed by qRT-PCR (SYBR green) **(C,D)** and Western blotting **(E,F)**, respectively. All experiments were repeated at least three times, and the data are expressed as mean ± SD. ^∗^*p* < 0.05, ^∗∗^*p* < 0.01 vs. Con or 0 h.

In contrast to the downregulation of nuclear receptors and their target genes, the expression of DEC1 was significantly upregulated in both primary hepatocytes and HepG2 cells (**Figure 3**). Among primary hepatocytes, the increase of DEC1 mRNA ranged from three to eightfold (**Figures 3A,B**). The magnitude of the increase in HepG2 cells was less and occurred in a concentration-dependent manner at both mRNA and protein levels (**Figures 3C–F**). Moreover, the maximal increase of DEC1 was detected at 12 h after IL-6 treatment (**Figures 3D,F**).

### IL-6 Differentially Regulates Nuclear Translocation of DEC1, PXR, and CAR

**FIGURE 4:**
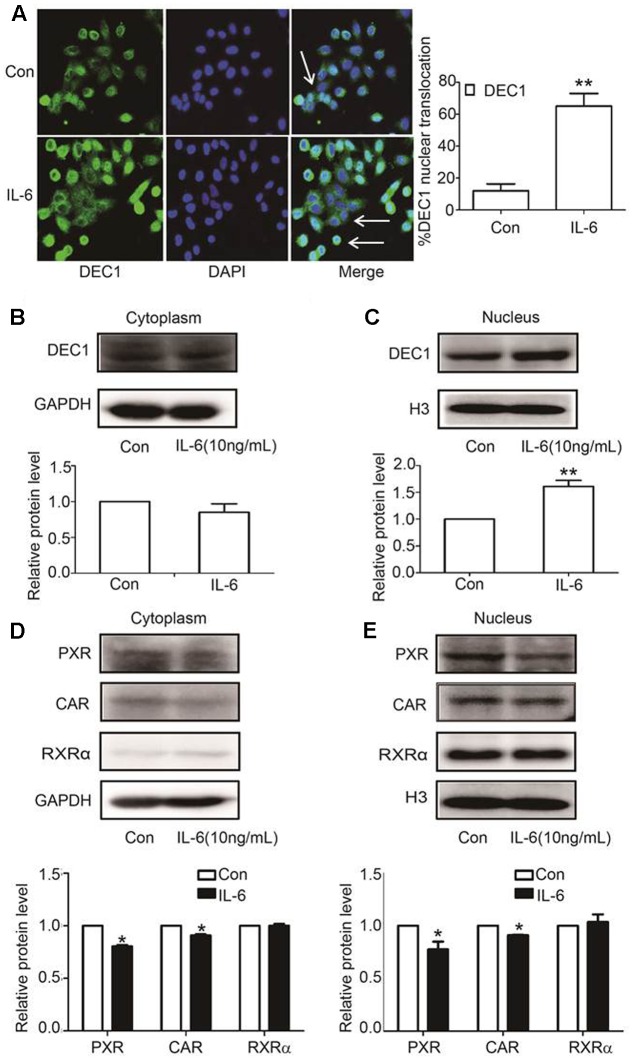
IL-6 induces DEC1 translocation. **(A)** Effect of IL-6 on the DEC1 translocation with immunofluorescence analysis. HepG2 cells were treated with IL-6 (10 ng/ml) for 24 h, and DEC1 protein translocation was detected by immunofluorescence analysis. Percentages of DEC1 nuclear translocation were determined by counting 100 to 150 cells in 3 or 4 non-overlapping fields. **(B,C)** Effect of IL-6 on DEC1 expression with plasmolysis. HepG2 cells were treated with IL-6 for 24 h. DEC1 or GAPDH cytoplasm protein and DEC1 or H3 nuclear protein expression were monitored by Western blotting. **(D,E)** Effect of IL-6 on PXR, CAR, and RXRα expression with plasmolysis. HepG2 cells were treated with IL-6 for 24 h, and PXR, CAR, RXRα cytoplasm and nuclear protein levels were monitored by Western blotting. All the experiments were repeated at least three times, and the data are expressed as mean ± SD. ^∗^*p* < 0.05, ^∗∗^*p* < 0.01 vs. Con.

DEC1, PXR, and CAR are transcription factors and next we tested whether IL-6 alter their nuclear translation. HepG2 cells were treated with IL-6 and then analyzed for the subcellular abundance with immunofluorescence staining. As shown in **Figure 4A**, DEC1 protein was significantly induced by IL-6 but the induction was primarily in the nucleus. Consistent with the immunofluorescence staining, nuclear DEC1 increased by as much as 60% but cytosolic DEC1 slightly decreased (**Figures 4B,C**). In contrast, comparable decreases of PXR and CAR were detected in both cytosolic and nuclear fractions in IL-6 treated cells (**Figures 4D,E**). Once again, no changes were detected on the subcellular abundance of RXRα.

### DEC1 Is a Common Suppressor for PXR, CAR and Their Target Genes in Response to IL-6

**FIGURE 5:**
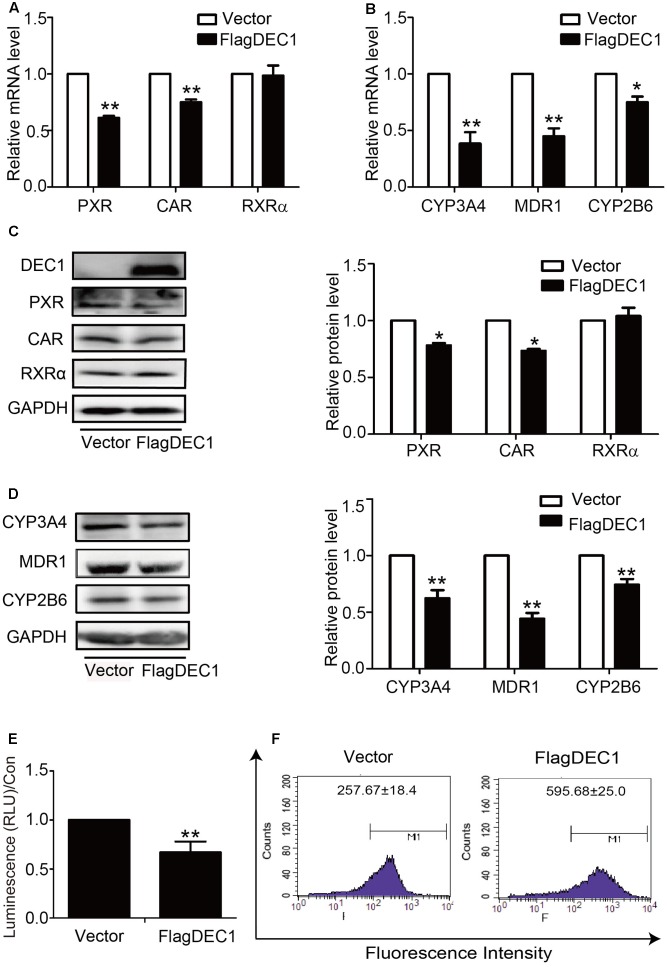
Overexpression of DEC1 inhibits the expression and function of PXR, CAR and their targets. HepG2 cells were transfected with the FlagDEC1 construct or the corresponding vector for 48 h. The levels of PXR, CAR, RXRα, CYP3A4, MDR1, and CYP2B6 were analyzed by qRT-PCR **(A,B)** and Western blotting **(C,D)**. **(E)** Overexpression of DEC1 inhibits the CYP3A4 activity. **(F)** Overexpression of DEC1 inhibits the efflux activity of MDR1. Representative histogram plots were provided with mean fluorescent intensity in the selected region of each treatment, as shown in the respective plot as the mean ± SD. To determine the transfection efficiency, cell lysates (10 μg) were analyzed for DEC1 using Western blotting. All experiments were repeated at least three times, and the data are expressed as mean ± SD. ^∗^*p* < 0.05, ^∗∗^*p* < 0.01 vs. vector (FlagCMV2).

To link IL-6 upregulation of DEC1 to a broader significance in the overall drug processing capacity, we performed DEC1 overexpression and knockdown for the effect on the expression of nuclear receptors and their target genes. With an exception of RXRα, overexpression of DEC1 decreased the expression of all genes tested. PXR, CAR, CYP2B6, CYP3A4, and MDR1 at both mRNA and protein levels (**Figures 5A–D**). Consistent with the downregulation, the activity of CYP3A4 and efflux activity of MDR1 also decreased significantly (**Figures 5E,F**). Once again, DEC1 overexpression did not change the level of RXRa (**Figures 5A,C**).

**FIGURE 6:**
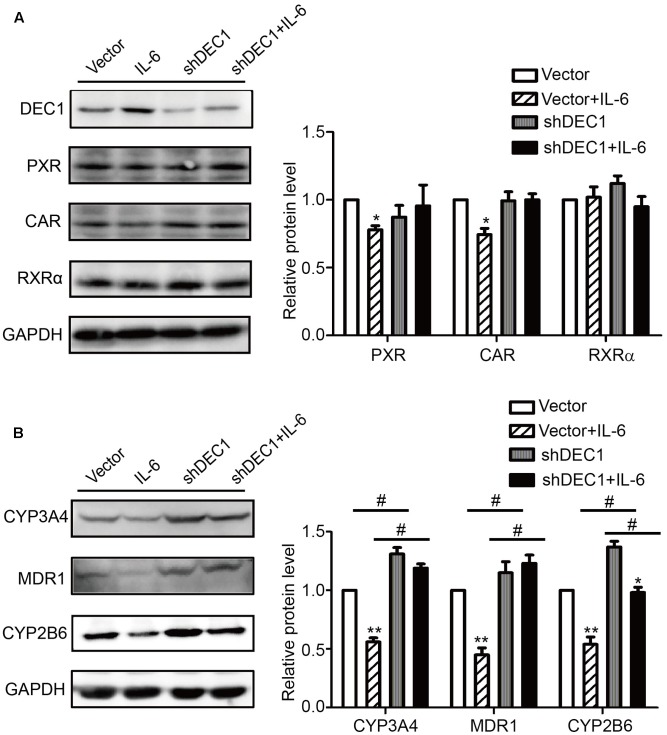
Knockdown of DEC1 reverses the decreases in PXR, CAR and their target genes induced by IL-6. **(A,B)** Effects of DEC1 knockdown on PXR, CAR, RXRα, CYP3A4, MDR1, and CYP2B6 protein levels induced by IL-6. HepG2 cells were transiently transfected with a mixture containing DEC1 shRNA construct or the corresponding vector. After a 72 h incubation, the transfected cells were treated with IL-6 (10 ng/ml) or PBS for 24 h, and the lysates (80 μg) were prepared and analyzed by using Western blotting. To determine the transfection efficiency, the cell lysates (40 μg) were analyzed for DEC1 using Western blotting. All the experiments were repeated at least three times, and the data are expressed as mean ± SD. ^∗^*p* < 0.05, ^∗∗^*p* < 0.01, a statistically significant decrease by IL-6 treatment (IL-6 treatment vs. without IL-6 treatment); ^#^*p* < 0.05, a statistically significant increase by DEC1 shRNA transfection (shRNA transfection vs. vector transfection).

To confirm the results with DEC1 overexpression, we tested DEC1 knockdown for the reversal effect. As expected, IL-6 decreased the expression of PXR, CAR, MDR1, CYP3A4, and CYP2B6 but not RXRα at both mRNA and protein levels (**Figure 6**). Importantly, the decrease was attenuated by DEC1 knockdown. It should be noted that that DEC1 knockdown alone significantly increased the expression of CYP3A4, CYP2B6, and MDR1 but not PXR or CAR (**Figure 6**).

### IL-6 Downregulation of PXR and CAR Is Achieved by Transrepression

**FIGURE 7:**
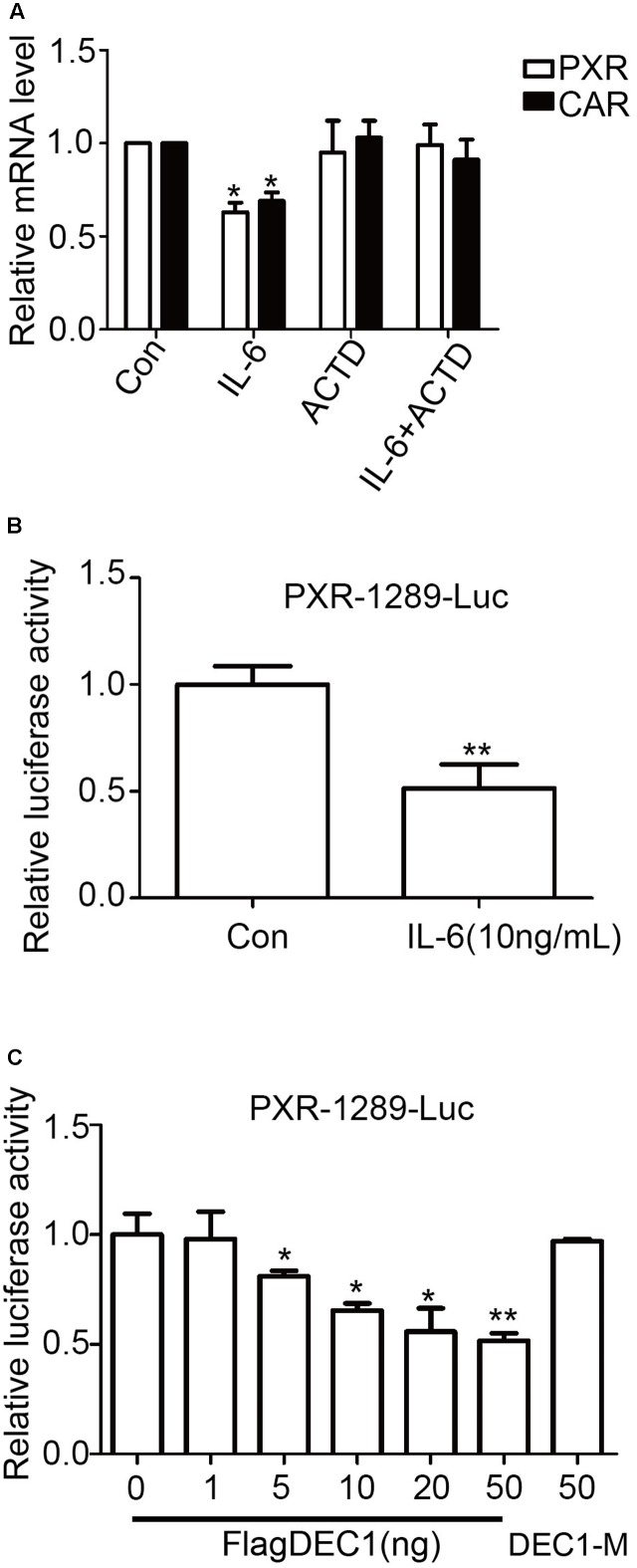
Transcriptional involvement in suppression of PXR and CAR induced by IL-6. **(A)** Effect of actinomycin D on suppression PXR and CAR mRNA. HepG2 cells were treated with 10 ng/ml IL-6 for 12 h in the absence or presence of 0.1 μM actinomycin D. Total RNA was prepared and analyzed for the PXR and CAR mRNA level. **(B)** The inhibition of the PXR-1289-Luc promoter reporter induced by IL-6. HepG2 cells were transiently transfected with a mixture containing 50 ng of PXR-1289-Luc, along with 5 ng of the Null-Renilla reniformis luciferase plasmid. The transfected cells were treated with 10 ng/ml IL-6 for 24 h. Luciferase activity was determined using the Dual-Luciferase reporter assay system. Reporter activity was normalized based on the Null-Renilla reniformis luminescence signal. **(C)** Overexpression of DEC1 represses the PXR promoter reporter. HepG2 cells were cultured in 48-well plates and transfected with PXR-1289-Luc (50 ng), DEC1 (1, 5, 10, 20, and 50 ng) or DEC1-M (50 ng) along with 5 ng of the Null-Renilla reniformis luciferase plasmid for 24 h. Cells were collected and analyzed for luciferase activity. The data were expressed as normalized luciferase activity (based on Null-Renilla reniformis luminescence signal). All the experiments were repeated at least three times, and the data are expressed as mean ± SD. ^∗^*p* < 0.05, ^∗∗^*p* < 0.01 vs. Con (PBS) or vector (FlagCMV2).

The comparable decrease of PXR and CAR at the mRNA and protein levels in response to IL-6 suggested that this proinflammatory cytokine suppressed the expression through transrepression, increased degradation of mRNA, or both. To gain initial insight, the effect of IL-6 on PXR and CAR mRNA expression was studied in the presence of actinomycin D (ActD), an inhibitor of RNA synthesis. HepG2 cells were treated with IL-6, ActD or both, and the levels of PXR and CAR mRNA were determined. As shown in **Figure 7A**, co-treatment with Act D abolished IL-6 downregulation of PXR and CAR, suggesting that IL-6 suppressed the expression of both nuclear receptors by transrepression. To test this possibility, cotransfection was performed with a PXR promoter reporter (PXR-1289-Luc). HepG2 cells were transfected with mixtures containing 50 ng of PXR-1289-Luc and 5 ng of Null-Renilla luciferase plasmid for 12 h. Transfected cells were then treated with IL-6 or PBS for 24 h. As shown in **Figure 7B**, treatment with IL-6 resulted in decreased reporter activity by as much as 45%. Importantly, cotransfection of DCE1 caused a comparable repression of this reporter (**Figure 7C**, 6 columns on the left). The repression was not detected with a DNA binding mutant of DEC1 (DEC1-M) (**Figure 7C**, 1 column on right).

### Stoichiometric Interaction of DEC1 with RXRa

**FIGURE 8:**
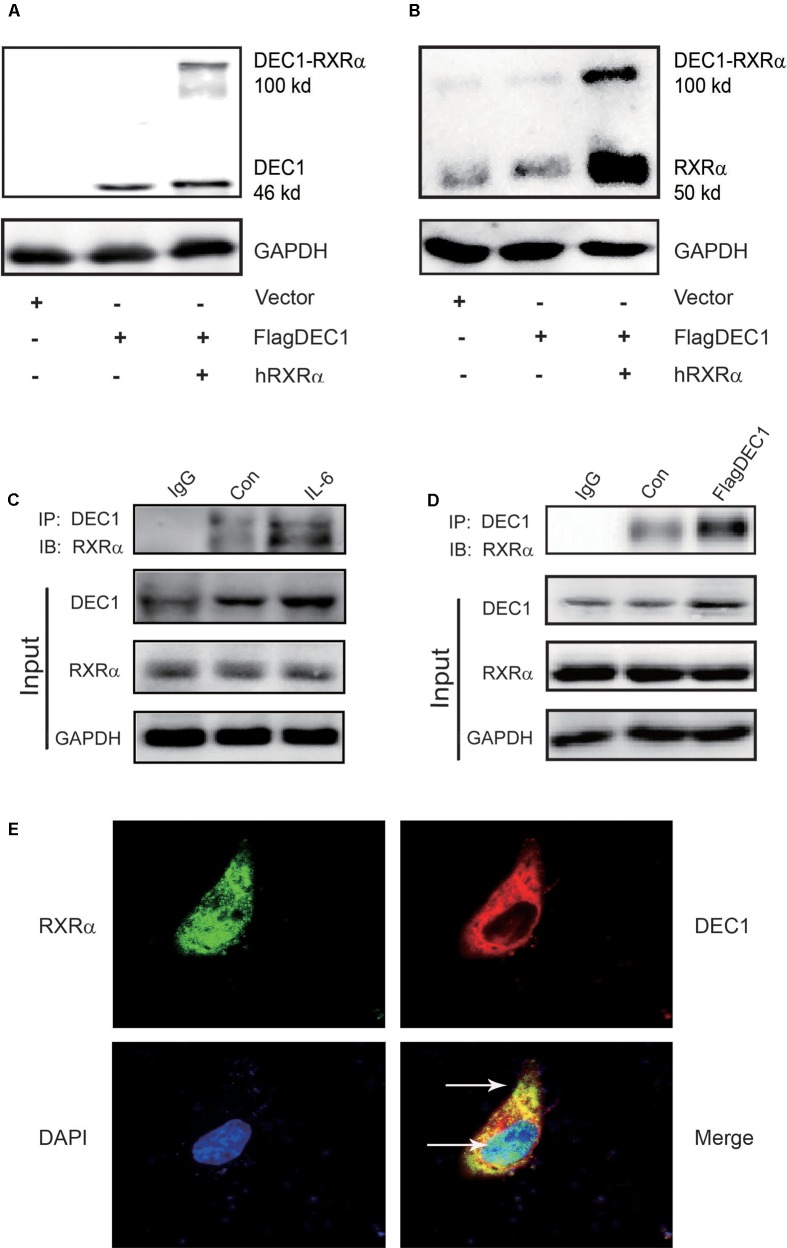
DEC1 combines with RXRαa molecular chaperone of nuclear receptors. **(A,B)** Protein–protein cross-linking experiments *in vivo*. HepG2 cells were transfected with the FlagDEC1 construct, RXRα or the corresponding vector for 48 h. Cell lysates (20 μg) were analyzed by Western blotting for the presence of crosslinked DEC1-RXRα. **(C,D)** Detection of DEC1 and RXRα by Co-Immunoprecipitation. Cells were treated with IL-6 (10 ng/ml) for 12 h, or transfected with FlagDEC1 construct, or the corresponding vector for 48 h. Cell lysates were incubated with an antibody against DEC1 or non-immune IgG. The immunoprecipitated complex was analyzed using antibodies against RXRα. The input was analyzed by Western blotting for DEC1, RXRα or GAPDH. **(E)** Co-localization of DEC1 and RXRα. HepG2 cells were transfected with FlagDEC1 construct and RXRα for 24 h. Immunofluorescence staining was performed by using antibodies against DEC1 (red), RXRα (green), and DAPI (blue). An over-lapping subcellular distribution (yellow) was presented in merged images.

We have shown that IL-6 downregulated PXR, CAR and their target genes. However, the magnitude of the downregulation of the target genes was greater than PXR or CAR (**Figure 1**). We have also shown that DEC1 was involved in the downregulation. Both PXR and CAR transactivate their target genes by dimerizing with RXRα (Ihunnah et al., 2011; Litwa et al., 2016). However, IL-6 caused little changes on the expression of RXRα (**Figures 1**, **2**). We next tested whether DEC1 interacts with RXRα. First, we performed intracellular cross-linking experiments. HepG2 cells were transfected with the corresponding vector, FlagDEC1, or both. The transfected cells were then fixed with 4% formalin. Cell extracts were prepared and analyzed by Western blotting. As expected, the antibody against DEC1 detected a protein with a molecular weight of ∼46 kDa in DEC1 transfected cells (**Figure 8A**). Importantly, the same antibody detected an extra protein band with a molecular weight of 100 kDa in DEC1/RXRα cotransfected cells (**Figure 8A**). The extra protein band has a molecular weight equal to the combined size of DEC1 (46 kDa) and RXRα (50 kDa) (**Figure 8A**). The antibody against RXRα detected the extra protein band as well (**Figure 8B**), suggesting that this band represented the DEC1-RXRα dimer.

Next, we performed co-immunoprecipitation with lysates from IL-6 treated or DEC1 transfected cells. In either case, the lysates were immunoprecipitated with anti-DEC1 antibody and the precipitates were detected by Western blotting with anti- RXRα antibody. As shown in **Figures 8C,D**, RXRα was present in the immunocomplex precipitated with anti-DEC1 antibody from IL-6 treated or DEC1 transfected cells. It appeared that DEC1 transfected cells produced a great presence of RXRα in the complex from DEC1 transfected than IL-6 treated cells. This DEC1-RXRα dimer was confirmed by dual-labeled immunofluorescence analyses in cells treated with IL-6 (**Figure 8E**).

### IL-6 Decreases PXR, CAR, and Their Targets and Increases STAR13 (DEC1) in Primary Mouse Hepatocytes

**FIGURE 9:**
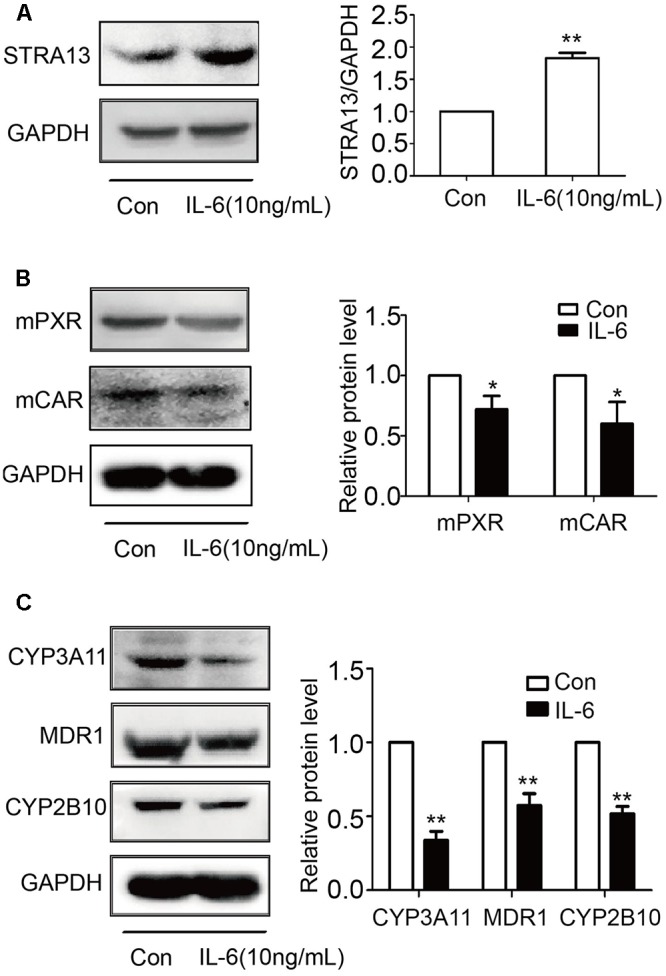
IL-6 increases STRA13 expression and decreases mPXR, mCAR, and their target genes in primary mouse hepatocytes. Primary mouse hepatocytes were treated with IL-6 (10 ng/ml) or PBS for 24 h. Cell lysates (40 μg) were prepared and subjected to Western blotting analyses with antibodies against STRA13 **(A)**, mPXR, mCAR **(B)**, CYP3A11, MDR1, CYP2B10 **(C)**, or GAPDH. All experiments were repeated from at least 6 mice (*n* = 6). Data are expressed as mean ± SD. ^∗^*p* < 0.05, ^∗∗^*p* < 0.01 vs. Con (PBS).

To examine whether mouse hepatocytes respond to IL-6 similarly as human hepatocytes and HepG2cells, mouse hepatocytes were treated with IL-6 (10 ng/ml) and lysates were analyzed for STAR13 (DEC1), PXR, CAR, and their targets by Western blotting. As shown in **Figure 9**, IL-6 significantly increased the level of STAR13 (DEC1) protein (**Figure 9A**). In contrast, the levels of PXR, CAR and their target genes (CYP3A11, CYP2b10, and MDR1) were decreased (**Figures 9B,C**). Once again, the expression of the target genes was decreased to a greater extent than that of PXR or CAR.

## Discussion

Inflammatory burden is a primary cellular event in many liver diseases, and IL-6 is a major proinflammatory cytokines (Azher et al., 2017; Prystupa et al., 2017). We and other investigators clarify that this very cytokine downregulated genes involved in drug elimination (Yang et al., 2007; Mehta et al., 2015; Kawauchi et al., 2016). In this study we used primary hepatocytes (human and mouse) as well as cell line and demonstrated that IL-6 induced DEC1, enhanced DEC1 interaction with RXRα and suppressed the expression of PXR, CAR and their target genes: CYP2B6, CYP3A4, and MDR1.

Induction of DCE1 plays a determinant role in IL-6 downregulation of PXR, CAR and their target genes. We have provided several lines of evidence that support this notion. IL-6 inversely regulated the expression of DEC1 over PXR, CAR and their target genes (**Figures 1**–**4**) with DEC1 being induced and the others being suppressed. In addition, overexpression of DEC1 caused similar changes as IL-6 in the expression profiles of these genes and knockdown of DEC1 reversed IL-6 effect. It should be noted that the knockdown of DEC1 alone increased the expression of PXR and CAR as well as their targets: CYP2B6, CYP3A4, and MDR1 (**Figures 6A,B**). It is likely that the constitutive expression of DEC1 (without IL-6 treatment) downregulates the expression of these genes (**Figures 5A–F**). Knockdown of DEC1 removes their constitutive downregulation.

DEC1 is a ligand-independent transcription factor and the suppression of PXR and CAR is likely achieved by transrepression in response to IL-6. As shown in **Figure 7**, the decreased expression of PXR and CAR in IL-6 treated cells was abolished by ActD, an RNA synthesis inhibitor (**Figure 7A**), pointing to an involvement of transcriptional regulation. In addition, both IL-6 and DEC1 repressed the PXR promoter reporter (**Figures 7B,C**). The repression was not detected with a DEC1 mutant lacking the DNA binding domain (**Figure 7C**).

In contrast to PXR and CAR, the expression of RXRα, a common dimerizing partner of both receptors, was not altered by IL-6 or DEC1 (**Figures 1C,D**, **2C,D**, **4D,E**, **5C**, **6A**). Instead, cross-linking and co-immunoprecipitation experiments detected interaction between DEC1 and RXRα (**Figures 8A–D**). It is likely that DEC1 interacts with free but not dimerized RXRα. As shown in **Figure 8A**, cross-linking experiment detected an extra protein band in cells cotransfected with DEC1 and RXRα. This band had a molecular weight of ∼100 kDa, equal to the combined molecular weight of DEC1 and RXRα. The DEC1-RXRα interaction likely provides an important mechanism by which the target genes of PXR and CAR as well as other RXRα-dimerizing partners are downregulated, at least in the presence of IL-6. In support of this functional importance, we have shown that PXR and CAR were consistently downregulated by IL-6 to a less extent than their target genes: CYP2B6, CYP3A4, and MDR1 (**Figures 1A–H**, **2A–F**, **9B,C**).

In summary, PXR and CAR are two master regulators of a wide range of genes involved in drug metabolism and transport. In this study, we have mechanistically linked induction of DEC1 to the suppressed expression of these nuclear receptors and their prototypical target genes in the presence of IL-6. This proinflammatory cytokine is elevated in many liver conditions: metabolic imbalance, viral infection and excessive alcohol consumption. Therefore, the findings reported in this study may have a broad implication, particularly related liver diseases with elevated inflammatory burden such as hepatitis and cirrhosis.

## Author Contributions

JY designed the research project. RN, YZ, SH, GH, BY, JY, and WL had full controlled the experiments, data analysis, and preparation of article. All authors were involved in planning the analysis and drafting the article. The final draft article was approved by all the authors.

## Conflict of Interest Statement

The authors declare that the research was conducted in the absence of any commercial or financial relationships that could be construed as a potential conflict of interest.

## Supplementary Material

FIGURE S1 Determine the different target proteins with corresponding positive controls.

## Abbreviations

CAR: constitutive androstane receptor
CYP2B6: cytochrome P450 2B6
CYP3A4: cytochrome P450 3A4
DEC1: differentiated embryonic chondrocyte-expressed gene 1
DMEM: Dulbecco’s modified Eagle’s medium
FBS: fetal bovine serum
FXR: farnesoid X receptor
GAPDH: glyceraldehyde-3-phosphate dehydrogenase
IL-6: interleukin-6
LXR: liver X receptor
MDR1: multidrug resistance 1
PXR: Pregnane X receptor
RAR: retinoic acid receptor
RXR α: retinoid X receptor-α
VDR: vitamin D receptor.
