# Supplementary figures and images for: Interleukin-6 Induces DEC1, Promotes DEC1 Interaction with RXRα and Suppresses the Expression of PXR, CAR and Their Target Genes
Source: Front Pharmacol. 2017 Nov 28;8:866. doi: 10.3389/fphar.2017.00866 (PMC5712319; doi:10.3389/fphar.2017.00866)

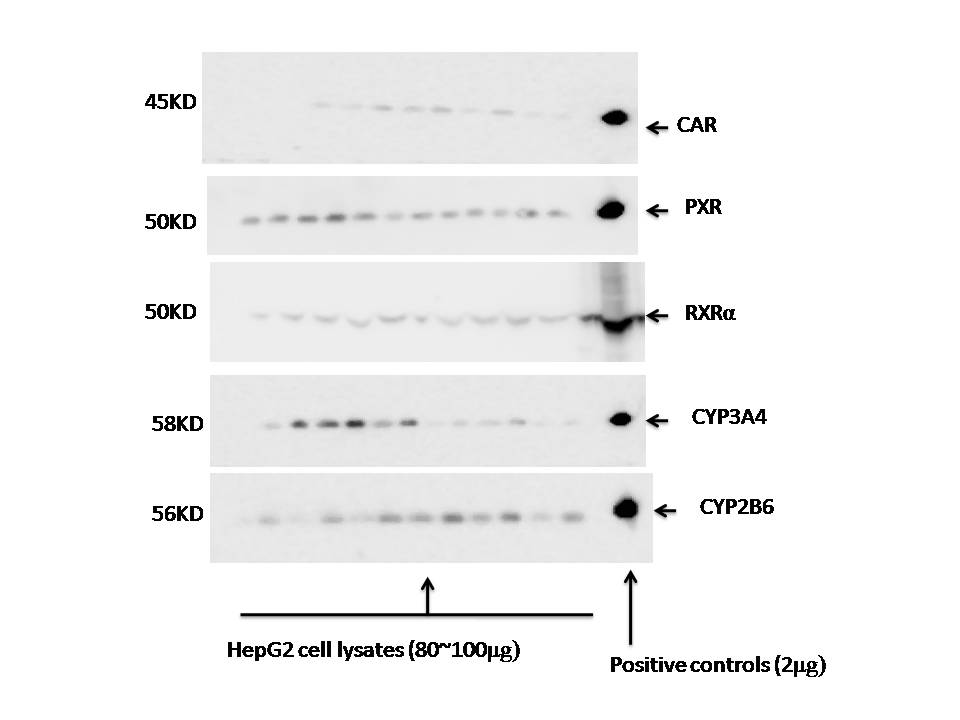

Supplement: FIGURE S1 — Determine the different target proteins with corresponding positive controls. [file Image_1.JPEG]
